# Development and validation of a questionnaire on radiation protection knowledge, attitudes, and practices among Moroccan dentists

**DOI:** 10.1002/acm2.14555

**Published:** 2024-11-05

**Authors:** Naoual Elmorabit, Majdouline Obtel, Mohamed Azougagh, Asmaa Marrakchi, Oum Keltoum Ennibi

**Affiliations:** ^1^ Laboratory of Research on Oral Biology and Biotechnology Faculty of Dental Medicine Mohammed V University Rabat Morocco; ^2^ Higher Institute of Nursing and Health Technology Ministry of Health and Social Protection Rabat Morocco; ^3^ Department of Public Health Faculty of Medicine and Pharmacy Mohammed V University Rabat Morocco; ^4^ National Graduate of Arts and Crafts (ENSAM) Mohammed V University Rabat Morocco; ^5^ Laboratory Health and Biology, Faculty of Sciences Ibn Tofail University Kenitra Morocco

**Keywords:** dentistry, knowledge, questionnaire, radiation protection, validity and reliability

## Abstract

**Purpose:**

This study aimed to develop and evaluate the validity and reliability of dentists' radiation protection knowledge, attitudes, and practices (DRP‐KAPs) questionnaire.

**Methods:**

This study was conducted using a stepwise approach. In the first step, items were generated to determine the relevant content and domains after a thorough literature review. In the second step, the content validity of the questionnaire was assessed by seven experts using face and content validity. The content validity index for relevance and clarity (I‐CVI, S‐CVI/Av, and S‐CVI/UA) and the content validity ratio (CVR) were performed. Then, the questionnaire was pre‐tested with 10 dentists for face validation. In the third step, reliability was assessed using internal consistency (Kuder‐Richardson‐20 (KR‐20) and Cronbach's alpha) and test–retest (Kappa and intraclass correlation coefficient [ICC]) methods by filling out the questionnaire by 100 dentists practicing in the Rabat‐Salé‐Kénitra region, in Morocco.

**Results:**

The finalized DRP‐KAPs questionnaire contains 41 items covering knowledge, attitudes and practices (KAPs). The I‐CVIs, S‐CVI/UA and S‐CVI/AV and CVR values of the 41 items were ≥0.86 for each item, ≥0.82, ≥0.97, and ≥0.71, respectively. With regard to internal consistency reliability, the KR‐20 coefficients for the knowledge and practice domains were 0.70 and 0.68, respectively, and the Cronbach alpha for the attitude domain was 0.73. The DRP‐KAPs questionnaire has good reliability with the ICC coefficients for attitude items ranging from 0.57 to 0.95 and Kappa coefficients for knowledge and practice items ranging from 0.64 to 1 and 0.77 to 1, respectively.

**Conclusion:**

The developed DRP‐KAPs questionnaire was found to be a noteworthy tool for assessing radiation protection among dentists, with acceptable internal consistency, good ICC and Kappa coefficients, and good content validity indices.

## 1 INTRODUCTION

Dental radiology has long been an important diagnostic tool in dentistry, especially with the increase in imaging modalities available today.^1^ This has enhanced diagnosis and treatment planning but it also carries a potential risk of unnecessary radiation exposure for both practitioners and patients.^2^ Although the doses used in routine dental radiography are relatively low, it is important to recognize that no exposure to ionizing radiation can be considered completely risk‐free. While the potential risk associated with these small radiation doses in dental radiography remains unclear, it is well‐established that any amount of ionizing radiation exposure can increase the likelihood of stochastic effects, such as cancer.^3^, ^4^, ^5^ Most epidemiological studies suggest a possible correlation between head and neck‐related tumors and exposure to dental x‐rays,^6^, ^7^ however, evidence for direct causation is still lacking.^8^, ^9^ Frequent dental x‐ray exams correlate directly with increased radiation exposure risks, which are particularly significant for pediatric patients who are more radiosensitive, especially girls. This heightened radiosensitivity makes children more vulnerable to long‐term effects such as radiation‐induced cancer, given their longer life expectancy compared to adults.^3^, ^9^, ^10^, ^11^ Dentists, managing both medical necessity and financial pressures, often rely on protocol‐based imaging, potentially leading to overprescription.^3^, ^12^

Thus, dentists must balance the potential risks and benefits of each x‐ray. This requires following ALARA (as low as reasonably achievable) principle during dentist routine work to ensure adequate justification with minimum and inevitable exposure.^13^, ^14^ To achieve this, all dental staff should be aware of the risks related to using x‐ray equipment, the recommended safety and precautionary measures, and the importance of compliance.^5^ Knowledge of radiation protection and its practice plays a crucial role in dentistry to protect patients, operators, and people around them.^15^ However, a review study revealed that dentists lacked knowledge and perception of how to manage radiation risks in dental practices.^16^ Furthermore, several studies showed that dentists' knowledge fails to meet required levels, which inadequately affects their attitudes and behaviors.^15^, ^17^, ^18^

Some knowledge, attitudes, and practices (KAPs) studies on dental radiation protection have used questionnaires that were not psychometrically assessed. It is important to note that in order to provide scientifically sound and clinically relevant findings, a questionnaire must be validated and present good psychometric properties, such as validity and reliability.^19^ The use of a reliable and valid tool increases the objectivity of findings and provides quantifiable outcomes that improve communication and generalizability.^20^ As there is currently no valid and reliable psychometric test available to evaluate compliance with radiation protection in dentistry in Morocco, this study aimed to develop and validate a questionnaire to assess dentist's radiation protection KAPs (DRP‐ KAPs).

## 2 METHODS

### 2.1 Study design and instruments

This cross‐sectional study aimed to examine dentists' radiation protection knowledge, attitudes, and practices (DRP‐KAPs) in the Rabat‐Salé‐Kenitra region of Morocco from April to June 2022. A questionnaire was used to collect data. All dentists employed in public, semi‐public, and private workplaces were targeted.

The study was carried out using a stepwise approach according to the methodology recommended by the literature^19^, ^20^, ^21^, ^22^ (a) item generation and construction, (b) testing of face and content validity, and (c) reliability.

- **Item generation**: A thorough literature review was conducted using several databases including Google Scholar, PubMed, Science Direct, Scopus, and Nature, as well as international guidelines^4^, ^5^, ^23^, ^24^ and national regulations. This review attempted to determine the pertinent content and domains necessary to appropriately represent the DRP‐KAPs.

The questionnaire's knowledge section was developed with a focus on radiation protection principles and regulations, radiation dose, and biological effects of ionizing radiation. The practice of dental radiography, radiographic equipment, and techniques best suited for operator and patient safety and exposure reduction were considered in the practice section of the questionnaire. Items exploring dentists' attitudes toward ordering x‐rays, asking patients about pregnancy, radiation protection measures, and methods for both patients and staff were included in the questionnaire's attitude section.

b. **Face and content validity**: An initial draft of 60 KAPs items was generated based on the literature review. After developing this first draft, the instrument was reviewed for face and content validity.^22^

Face validity is used to check how well questionnaire items (at face value) reflect the construct to be measured, in terms of wordings, structures, orderliness, and scoring formats.^25^

In order to test face and content validity, a group of at least 5–15 experts is required.^26^ Seven experts have assessed the face and content validity of the DRP‐KAPs questionnaire. Experts on the panel were carefully selected for their expertise and significant contributions to the field of dentistry and radiation protection research and evaluation. The panel included three experienced dentists, each with over a decade of practical experience—a professor specializing in periodontology, an orthodontist, and a general practitioner. This dental expertise was complemented by an epidemiologist versed in public health dynamics, as well as two distinguished professors—one specializing in biomedical engineering with a background in nuclear physics and radiation safety, and another in medical physics. Additionally, the panel included a radiation protection officer specializing in medical imaging services. The selection was made to ensure a thorough understanding of the topic and to represent a variety of viewpoints and expertise within the field. The experts evaluated the face validity of the items in terms of content, simplicity, perceptibility, and grammar, and any necessary changes were made.

It is strongly advised to apply content validity in the design of the new instrument.^27^ Content validity pertains to the ability of a questionnaire to adequately cover all relevant topics of the study construct (concept) to be measured.^25^

Various methods are used in the literature to confirm quantitative content validity, such as the content validity index (CVI) and content validity ratio (CVR).^25^, ^28^

In this step, each question's CVI was calculated. Experts were requested to rate questionnaire items in terms of clarity and relevancy on a 4‐point Likert scale (1 = not clear, or not relevant, 2 = Item needs major revision to be clear or relevant, 3 = Item needs minor revision to be clear or relevant and 4 = very clear, or very relevant) as done in earlier studies.^29^, ^30^ This four‐point scale was also used to prevent a neutral or ambivalent midpoint.^31^

The CVI for the questionnaire's clarity and relevancy was tested using the item content validity index (I‐CVI) and scale content validity index (S‐CVI). The I‐CVI index was calculated by dividing the number of experts who rated the item as 3 or 4 by the total number of experts.^31^

To determine the S‐CVI index, both the average method and the universal agreement method were used.^32^ The average scale‐level content validity index (S‐CVI/Ave) is calculated by adding up all of the item's I‐CVIs and dividing the result by the total number of items.^33^ The universal agreement method (S‐CVI/UA) is defined as the proportion of items on an instrument that achieved a rating of 3 or 4 by all the content experts.^31^

The questionnaire item was retained if the item I‐CVI for subject relevancy and clarity was greater than 0.79. If the I‐CVI value was in the range of 0.70‐0.79, the item was revised. Items with an I‐CVI value less than 0.70 were rejected.^30^, ^34^ Values of S‐CVI/UA ≥ 0.80 and S‐CVI/Ave ≥ 0.90 indicated excellent content validity.^30^, ^31^, ^34^

The CVR is computed for each item using Lawshe's method (1975).^27^, ^35^ The panel was asked to rate each item as “essential,” “useful but not essential,” or “not necessary”. The CVR has been formulated as follows: CVR = (Ne—*N*/2)/(*N*/2), where Ne is the number of content experts indicating “essential” and *N* is the total number of content experts (Lawshe 1975). Lawshe (1975) has provided a table for determining the minimum CVR value required for an item to be considered acceptable, based on the number of experts. In this study, the minimum significant CVR with 7 panellists were 0.99 per item defined, according to Lawshe's table.^35^ Based on the expert opinions, the items with very low CVR (<0) were removed. The items with 0 ≤CVR (i.e., half or more of the experts rated these items as “essential”), but which revealed a high level of relevancy and clarity, were retained in the questionnaire, revised, and sent to the experts. After the second review, the CVR, S‐CVI/Ave, S‐CVI/UA and I‐CVI were computed.^36^

In light of the data analysis's findings and the expert panel's recommendations, some changes and revisions were made. Subsequently, the questionnaire was sent to 10 dentists as a target group to test for clarity, item understanding, and item wording.^25^ It was also assessed how long it took on average to complete the questionnaire. These 10 dentists were excluded from the following phases of the study. Based on the feedback and suggestions, minor adjustments have been incorporated to further improve the clarity of the questionnaire.

c. **Reliability**: Reliability was checked using test–retest and internal consistency. Questionnaires were filled out by 100 Moroccan dentists practicing in the Rabat‐Salé‐Kénitra region, encompassing both the Public Dental Health Service and private practice, and including generalists and specialists involved in dental radiography.^37^, ^38^ Internal consistency was evaluated to determine the extent to which all scale items measured the same underlying construct or characteristic.^39^ To determine internal consistency, the Kuder‐Richardson‐20 (KR‐20) coefficient was used for the knowledge and practice items, as they are scored dichotomously.^30^, ^36^ The Cronbach's alpha coefficient was applied to attitude domain items with cutoff value of ≥ 0.70.^19^, ^32^, ^40^ KR‐20 values greater than 0.6 indicated test homogeneity and item integrity.^41^

The test–retest method was used to measure the questionnaire's stability.^42^ In this step, the reliability of the instrument was assessed after completing the redistributed questionnaires by 50 out of 100 dentists of the same population over a two‐week interval. To determine the correlated reliability agreement the Kappa index was used for knowledge and practice (nominal variables) domains while the intra‐class correlation (ICC) index was applied to attitude domains (ordinal variables).^19^, ^43^, ^44^

The possible values of Kappa range from ‐1 to 1, with a typical value between 0 and 1. A value of 1 is considered a perfect agreement, while a value of 0 indicates an agreement no better than would be expected by chance and a negative Kappa would indicate an agreement worse than that expected by chance.^43^, ^44^ The proposed standards for the Kappa statistic's strength of agreement are as follows: 0 or lower = poor, 0.01–0.20 = slight, 0.21–0.40 = fair, 0.41–0.60 = moderate, 0.61–0.80 = substantial, and 0.80–1.0 = almost perfect.^43^, ^44^, ^45^ The reliability interpretation for the attitude domain based on the ICC values was classified as follows: “poor reliability” (ICC < 0.5), “moderate reliability” (0.5 ≤ICC < 0.75), “good reliability” (0.75 ≤ICC < 0.9)and “excellent reliability” (ICC ≥0.9).^45^, ^46^

## 3 RESULTS

### 3.1 Participant characteristics

**FIGURE 1:**
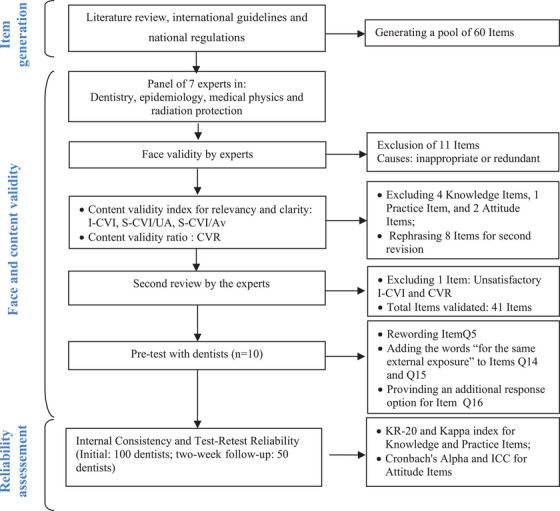
Flowchart describing the steps followed to develop and validate the DRP‐KAPs questionnaire. DRP‐KAPs, dentists' radiation protection knowledge, attitudes, and practices.

A total of 100 dentists in the Rabat‐Salé‐Kenitra region of Morocco were recruited for this study. Females accounted for 65% and males 35% of the study population. 37% were younger than 29, 34% were aged 30–39, 18% were aged 40–49, and the remaining dentists’ cohort was aged 50 or older (11%). The experience in dental practice was seen to be less than 10 years for 65% of participants. 51% of the dentists were in general dental practice and 49% worked as specialists in different dental fields. Of all participants, 53% worked in private practice and 47% practiced at public dental service. 53.6% of the participants had received continuing education in dental radiation protection. The process of questionnaire development and validation is summarised in Figure 1.

### 3.2 Face, content validity, and pre‐test phase

Initially, 60‐item questions were framed. Among these, 11 items were removed after the Face Validity assessment by experts, because they were either irrelevant or redundant. Thus, only 49 items remained and were subject to quantitative content validity. Of these 49 items, 17 pertained to radiation protection knowledge, 13 were related to practices, and 19 focused on attitudes.

Quantitative content validity assessed by 7 experts indicated that changes had to be made to the questionnaire. 4 items of knowledge dimension, 1 item of practice dimension, and 2 items of attitude dimension were removed from the questionnaire according to the experts due to low CVIs (relevancy and clarity of items) and CVRs. (Table 1)

**TABLE 1:** Expert panel evaluation of the items' I‐CVI and CVR (1st time).

Items		Relevancy I‐CVI	Clarity I‐CVI	CVR	Interpretation
Knowledge domain					
Q1	Awareness of radiation protection measures?	1.00	1.00	1.00	Retained
Q2	Awareness of ALARA principle?	1.00	1.00	1.00	Retained
Q3	Knowledge of the Moroccan radiation regulatory agency?	1.00	1.00	1.00	Retained
Q4	Awareness of international radiation protection recommendations in dentistry?	1.00	1.00	1.00	Retained
Q5	Awareness of the dentists' obligation to adhere to safety, usage, and maintenance instructions for x‐ray equipment?	1.00	1.00	1.00	Retained
Q6	Awareness of the dentist's obligation to establish a quality control plan for x‐ray equipment?	1.00	0.86	1.00	Retained
Q7	Annual radiation dose limit for a dentist in millisievert?	1.00	1.00	0.43	Rephrased
Q8	Dental x‐rays are harmful?	1.00	1.00	1.00	Retained
Q9	Awareness of stochastic and deterministic effects of radiation exposure?	0.57	0.43	0.71	Deleted
Q10	x‐rays are reflected off the walls of the room?	0.28	0.57	−0.43	Deleted
Q11	Awareness of the usefulness of collimation and filters in dental radiography?	0.57	0.71	0.14	Deleted
Q12	Any radiation exposure brings a possibility of stochastic effects, such as cancer induction?	1.00	0.86	1.00	Retained
Q13	The risk of cancer induction is cumulative over a lifetime for low‐dose exposures?	1.00	1.00	0.71	Rephrased
Q14	Knowledge of the most radiosensitive organs or tissues?	1.00	1.00	1.00	Retained
Q15	Pediatric patients and developing fetus are more sensitive/vulnerable to ionizing radiation?	0.29	0.57	0.43	Deleted
Q16	Children's radiation dose is higher than that of an adult?	1.00	1.00	1.00	Retained
Q17	Awareness that the risk of cancer induction in children is two to three times higher than in adults?	0.86	1.00	0.43	Rephrased
Practices domain					
Q18	Number of intraoral radiographs taken/prescribed per week	1.00	1.00	1.00	Retained
Q19	Number of extraoral radiographs taken/prescribed per week	1.00	1.00	1.00	Retained
Q20	Type of intraoral image receptor	0.86	1.00	1.00	Retained
Q21	Type of film processing used	0.71	0.86	0.14	Rephrased
Q22	Average exposure times used for intra‐oral x‐rays	0.57	0.71	0.43	Deleted
Q23	The peak kilovoltage of intraoral equipment	0.86	1.00	1.00	Retained
Q24	Type of collimator used in x‑ray unit	1.00	1.00	1.00	Retained
Q25	Types of intraoral cone used	1.00	1.00	0.71	Rephrased
Q26	Holding of film during exposure	1.00	1.00	1.00	Retained
Q27	Technique used for taking intraoral periapical radiographs (IOPAR)	1.00	1.00	0.43	Rephrased
Q28	Position taken during intra‐oral exposure	1.00	1.00	1.00	Retained
Q29	Distance from the x‐ray tube during intraoral exposure	1.00	1.00	1.00	Retained
Q30	Angle of the x‐ray tube during exposure	1.00	0.86	1.00	Retained
Attitudes domain					
Q31	Prescribing radiographs to patients after a clinical examination	1.00	1.00	1.00	Retained
Q32	Prescribing radiographs based on the patient's history	0.86	1.00	1.00	Retained
Q33	Prescribing radiographs routinely	1.00	1.00	1.00	Retained
Q34	Request radiographs from previous dentist to evaluate a new patient	0.86	1.00	0.71	Rephrased
Q35	Prescribing/performing intraoral radiography to evaluate a new patient	1.00	1.00	1.00	Retained
Q36	Prescribing/performing panoramic radiography to evaluate a new patient	1.00	1.00	1.00	Retained
Q37	Prescribing radiographs on request by third parties	1.00	0.86	1.00	Retained
Q38	Explaining the risks/benefits of irradiation to patients before the imaging procedure	1.00	1.00	0.43	Rephrased
Q39	Patients ask questions about radiation protection	0.29	0.42	−0.14	Deleted
Q40	Asking patients about pregnancy before the imaging procedure	1.00	1.00	1.00	Retained
Q41	Prescribing/performing dental imaging on pregnant patients	1.00	1.00	1.00	Retained
Q42	Wearing a lead apron to protect against x‐rays	1.00	1.00	1.00	Retained
Q43	Using rectangular collimator to protect against x‐rays	1.00	0.86	1.00	Retained
Q44	Using of the lowest exposure setting as possible to protect against x‐rays	0.86	0.86	1.00	Retained
Q45	Standing behind a protective barrier to protect against x‐rays	1.00	0.86	1.00	Retained
Q46	Using a lead apron to protect patient against x‐rays	1.00	1.00	1.00	Retained
Q47	Using a thyroid shield to protect patient against x‐rays	1.00	1.00	1.00	Retained
Q48	Wearing a dosimeter while working	1.00	1.00	1.00	Retained
Q49	Keeping a radiographic record / logbook in dental practice	0.14	0.29	0.43	Deleted

Abbreviations: CVR, content validity ratio; I‐CVI, items content validity index.

According to Lawshe,^35^ questions with a CVR > 0.99 were maintained in the questionnaire. In addition, items with a CVR < 0.99 that were not rated “unnecessary” by any of the experts and had an appropriate CVI (relevancy and clarity) underwent a second revision to enhance CVR scores.

Thirty‐four questions/items were retained with appropriate CVI and CVR scores and eight questions/items were modified due to the high CVI scores and low CVR scores and underwent a second revision (Tables 1 and 2).

**TABLE 2:** Expert panel evaluation of I‐CVI, S‐CVI, and CVR (2nd time).

Items		Relevancy I‐CVI	Clarity I‐CVI	CVR	Decision
Knowledge domain					
Q7	Annual radiation dose limit for a dentist in millisievert?	1.00	1.00	0.71	Retained
Q13	The risk of cancer induction may be cumulative over a lifetime for low‐dose exposures?	1.00	1.00	1.00	Retained
Q17	Awareness that the risk of cancer induction in children may be two to three times higher than in adults?	0.86	1.00	1.00	Retained
Practices domain					
Q21	Type of film processing used	0.57	0.71	0.43	Deleted
Q25	Types of intraoral cone used	1.00	1.00	1.00	Retained
Q27	Technique used for taking intraoral periapical radiographs (IOPAR)	1.00	1.00	0.71	Retained
Attitudes domain					
Q34	Request radiographs from previous dentist to evaluate a new patient	0.86	1.00	1.00	Retained
Q38	Explaining the risks/benefits of irradiation to patients before the imaging procedure	1.00	1.00	0.71	Retained
S‐CVI/UA					
Knowledge domain		0.92	0.85		
Practices domain		0.82	0,91		
Attitudes domain		0.82	0.76		
Total S‐CVI/UA		0.85	0.84		
S‐CVI/Ave					
Knowledge domain		0.99	0.98		
Practices domain		0.97	0.99		
Attitudes domain		0.97	0.97		
Total S‐CVI/Ave		0.976	0.98		

Abbreviations: CVR, content validity ratio; I‐CVI, items content validity index; S‐CVI/Ave, scale content validity index/average; S‐CVI/UA, scale content validity index/universal agreement.

After the second expert review, one additional item (Q21) was removed due to unsatisfactory item I‐CVI and CVR values. As a result, the questionnaire was approved with a total of 41 items: 13 knowledge items, 11 practice items, and 17 attitude items. the I‐CVI, S‐CVI/UA, S‐CVI/Ave, and CVR values of these 41 items were as follows: I‐CVI ≥0.86 for each item, S‐CVI/UA ≥0.82, S‐CVI/Ave ≥0.97 and CVR ≥0.71. (Tables 1 and 2).

The pre‐testing study revealed a few minor problems with the wording and presentation of the KAPs radiation protection questions. Respondents indicated that they struggled with some of the questions and needed to read them several times to fully comprehend them. Therefore, minor modifications were made. For example, Item Q5 was rephrased from “Awareness of the requirement to comply with instructions relating to the safety, use, and maintenance of x‐ray equipment” to “Awareness of the dentists' obligation to adhere to safety, usage, and maintenance instructions for x‐ray equipment” for clarity and simplicity.

Furthermore, for Item Q14 (Q16 in the first version of the questionnaire) the phrase “for the same external exposure” was added to the statement “children's radiation dose is higher than that of an adult.” Similarly, for Item Q15 (Q17 in the first version of the questionnaire) the phrase “for the same external exposure” was added to the statement “The risk of cancer induction in children is two to three times higher than in adults.” An additional response option “self‐developing films” was also added to Item Q16 (Q20 in the original version). It took less than 20 min for all participants to answer and comment on all questions.

### 3.3 Internal consistency and test–retest reliability

Once the final version of the 41‐item DRP‐KAPs had been developed, the questionnaire's psychometric properties were examined by administering it to the 100 study participants. Table 3 shows the reliability results for the knowledge and practice domains of the DRP‐KAPs questionnaire. The KR‐20 values for the knowledge and practice domains were 0.70 and 0.68, respectively. In the attitude domain, the Cronbach's alpha was 0.73 (Table 4). This indicates that the questions in each domain are well‐connected.

**TABLE 3:** Internal consistency reliability(KR‐20) and Kappa values (k) for knowledge and practice items.

Items		Internal consistency KR‐20 if item deleted	Internal consistency KR‐20	Kappa	*p*‐Values	Classification
**Knowledge domain**			0.70	0.82	<0.0001	Almost perfect
Q1	Awareness of radiation protection measures?	0.697		1.00	<0.0001	Almost perfect
Q2	Awareness of ALARA principle?	0.678		0.92	<0.0001	Almost perfect
Q3	Knowledge of the Moroccan radiation regulatory agency?	0.683		0.82	<0.0001	Almost perfect
Q4	Awareness of international radiation protection recommendations in dentistry?	0.673		0.786	<0.0001	Substantial
Q5	Awareness of the dentists' obligation to adhere to safety, usage, and maintenance instructions for x‐ray equipment?	0.698		0.919	<0.0001	Almost perfect
Q6	Awareness of the dentist's obligation to establish a quality control plan for x‐ray equipment?	0.702		0.905	<0.0001	Almost perfect
Q7	Annual radiation dose limit for a dentist in millisievert?	0.678		0.834	<0.0001	Almost perfect
Q8	Dental x‐rays are harmful?	0.703		0.819	<0.0001	Almost perfect
Q9	Any radiation exposure brings a possibility of stochastic effects, such as cancer induction	0.699		0.778	<0.0001	Substantial
Q10	The risk of cancer induction may be cumulative over a lifetime for low‐dose exposures	0.678		0.774	<0.0001	Substantial
Q11	Knowledge of the most radiosensitive organs or tissues?	0.688		0.64	<0.0001	Substantial
Q12	Children's radiation dose is higher than that of an adult for the same external exposure?	0.655		0.752	<0.0001	Substantial
Q13	Awareness that the risk of cancer induction in children may be two to three times higher than in adults, for the same external exposure?	0.655		0.781	<0.0001	Substantial
**Practices domain**			0.679	0.90	<0.0001	Almost perfect
Q14	Number of intraoral radiographs taken/prescribed per week	0.631		1.00	<0.0001	Almost perfect
Q15	Number of extraoral radiographs per week	0.619		1,00	<0.0001	Almost perfect
Q16	Type of intraoral image receptor	0.651		1,00	<0.0001	Almost perfect
Q17	The peak kilovoltage of intraoral equipment?	0.677		0.806	<0.0001	Almost perfect
Q18	Type of collimator used in x‑ray unit	0.682		0.847	<0.0001	Almost perfect
Q19	Types of intraoral cone used	0.657		0.922	<0.0001	Almost perfect
Q20	Holding of film during exposure	0.683		0.911	<0.0001	Almost perfect
Q21	Technique used for taking intraoral periapical radiographs (IOPAR)	0.649		0.866	<0.0001	Almost perfect
Q22	Position taken during intra‐oral exposure	0.625		0.958	<0.0001	Almost perfect
Q23	Distance from the x‐ray tube during intraoral exposure	0.667		0.879	<0.0001	Almost perfect
Q24	Angle of the x‐ray tube during exposure	0.675		0.767	<0.0001	Substantial

*Note*: *p*‐value < 0.05.

Abbreviation: KR‐20, Kuder‐Richardson‐20.

**TABLE 4:** Internal consistency reliability and ICC for attitude items.

Items		Internal Consistency Cronbach's Alpha	Cronbach's Alpha if item deleted	ICC	95% CI	Classification
	Attitude domain	0.73		0.76		Good
Q25	Prescribing radiographs to patients after a clinical examination		0.73	0.63	(0.43; 0.77)	Moderate
Q26	Prescribing radiographs based on the patient's history		0.71	0.66	(0.47; 0.79)	Moderate
Q27	Prescribing radiographs routinely		0.718	0.83	(0.72; 0.90)	Good
Q28	Request radiographs from previous dentist to evaluate a new patient		0.694	0.75	(0.61; 0.85)	Good
Q29	Prescribing/performing intraoral radiography to evaluate a new patient		0.706	0.57	(0.36; 0.73)	Moderate
Q30	Prescribing/performing panoramic radiography to evaluate a new patient		0.737	0.78	(0.64; 0.87)	Good
Q31	Prescribing radiographs on request by third parties		0.705	0.81	(0.68; 0.89)	Good
Q32	Explaining the risks/benefits of irradiation to patients before the imaging procedure		0.718	0.63	(0.44; 0.77)	Moderate
Q33	Asking patients about pregnancy before the imaging procedure		0.72	0.77	(0.63; 0.87)	Good
Q34	Prescribing/performing dental imaging on pregnant patients		0.71	0.81	(0.69; 0.89)	Good
Q35	Wearing a lead apron to protect against x‐rays		0.699	0.86	(0.76; 0.92)	Good
Q36	Using rectangular collimator to protect against x‐rays		0.735	0.85	(0.75; 0.91)	Good
Q37	Using of the lowest exposure setting as possible to protect against x‐rays		0.722	0.70	(0.53; 0.82)	Moderate
Q38	Standing behind a protective barrier to protect against x‐rays		0.705	0.83	(0.72; 0.90)	Good
Q39	Using a lead apron to protect patient against x‐rays		0.726	0.89	(0.82; 0.94)	Good
Q40	Using a thyroid shield to protect patient against x‐rays		0.733	0.66	(0.47; 0.79)	Moderate
Q41	Wearing a dosimeter while working		0.72	0.95	(0.93; 0.97)	Excellent

Abbreviations: CI, confidence interval; ICC, intraclass correlation coefficient.

To assess test–retest reliability, 50 dentists completed the questionnaire two weeks after the first survey. Most knowledge items attained substantial to almost perfect Kappa value (0.64 ≤ Kappa ≤ 1). Kappa values for practice items ranged from 0.77 to 1 (Table 3), showing substantial to almost perfect test–retest agreement. For all knowledge and practice items, agreement was significantly better than would be expected by chance alone (*p*‐value <0.0001).

Intraclass correlation coefficient (ICC) assessment of the attitude domain showed that reliability was moderate for six items (0.57 ≤ ICC ≤ 0.70), good for 34 items (0.75 ≤ ICC ≤ 0.89), and excellent for one item (ICC = 0.95). On the whole, the questionnaire showed good test–retest reliability (Table 4).

## 4 DISCUSSION

The purpose of this study was to create and present a psychometric assessment of the DRP‐KAPs questionnaire. To the best of our knowledge, this is the first study to offer a validated tool for assessing radiation protection KAPs among dentists in Morocco.

The availability of a validated DRP‐KAPs questionnaire is particularly important for the development of interventions aimed to improve knowledge and to change attitudes and practices toward the use of ionizing radiation in dentistry.

The psychometric evaluation of this questionnaire provided good evidence of its validity and reliability in assessing KAPs in dental radiation protection. Overall, the reliability and content of the results were satisfactory.

The DRP‐KAPs questionnaire is designed to improve radiation protection in dental clinical practice and can also be used in an educational context. The aim of this instrument is to assess the level of knowledge and compliance with radiation protection guidelines among dentists, to better understand their current practices, to identify needs for improvement, and to provide a basis for further research.

The DRP‐KAPs questionnaire contains 41 items covering KAPs domains. Acceptable cut‐offs were met by I‐CVIs (for item relevancy and clarity), S‐CVI/AV, S‐CVI/UA, and CVR in all 41 questionnaire items.^27^, ^29^, ^31^

Moreover, reliability testing was conducted to determine the internal consistency and reproducibility. The internal consistency reliability of the 13 items relating to the knowledge domain and 11 practice Items had KR‐20 coefficient values of 0.70 and 0.68, respectively, which were acceptable.^47^, ^48^ The internal consistency of the 17 attitude items was further ensured by a satisfactory Cronbach's alpha value (0.73) indicating adequate internal uniformity.^19^, ^32^, ^40^

The current study's results showed that the DRP‐KAPs questionnaire has good reliability in terms of ICC and Kappa coefficients, with ICC coefficients for attitude items ranging from 0.57 to 0.95 and Kappa coefficients for knowledge and practice items ranging from 0.64 to 1 and 0.77 to 1, respectively. The Retest reliability of the questionnaire met psychological measurement standards and showed good stability.^19^, ^44^, ^45^ As the reliability values were deemed acceptable, there was no need to exclude any items.

In comparison to similar studies that have aimed to validate Radiation Protection KAPs instruments among dentists or other healthcare professionals, the results of this study demonstrate notable strengths: good content validity indices, satisfactory ICC and Kappa coefficients, and acceptable internal consistency.

Previous research on Radiation Protection KAPs among dentists has exhibited various limitations, including the measurement of variables only categorically, lack of disclosure regarding validation processes, and focusing on individual dimensions of KAPs rather than jointly assessing them. These limitations have hindered the comprehensive understanding and evaluation of dentists' behaviors and perceptions related to radiation safety.^15^, ^17^, ^18^, ^49^

Fakhar et al.^40^ designed a standard questionnaire to assess the knowledge and attitudes of medical students regarding radiation protection. The amounts of I‐CVI and S‐CVI were in the excellent range (≥ 83.3% and 98.33%, respectively). In addition, internal consistency showed good results for all three concepts (basic knowledge = 0.793, practical knowledge = 0.823, and attitude = 0.822). The questions were focused on knowledge domains by covering both basic and practical aspects, and the attitude domain of medical radiation protection.^40^ Additionally, a healthcare professional knowledge of radiation protection (HPKRP) self‐evaluation scale was developed and psychometrically tested by Schroderus‐Salo et al.^22^ to measure the level of radiation protection knowledge possessed by healthcare professionals who deal with radiation in a clinical setting. The I‐CVI ranged from 0.66 to 1, the S‐CVI was 0.83 and the Cronbach's alpha was 0.98.^22^

On the other hand, a validation study by Ramírez et al.^50^ on knowledge in radiological protection of undergraduate and postgraduate students in dentistry reported good results. The reliability showed an ICC between 0.697 and 0.729 and a Cronbach's alpha of 0.727. These findings align closely with the current study's outcomes, reinforcing the questionnaire's efficacy in assessing radiation protection competencies within the dental education context. Furthermore, the study by Alavi et al.^51^ on the content validity and internal consistency reliability of a medical radiation workers' KAPs questionnaire also demonstrated acceptable results, with CVR and CVI values ranging from 0.61 to 0.76 and 0.77 to 0.93, respectively, and a Cronbach's alpha of 0.92 for all items.

Despite the differences in the contexts and target populations, the studies conducted by Fakhar et al.,^40^ Schroderus‐Salo et al.,^22^ Ramírez et al.^50^ and Alavi et al.^51^ converge on a common finding: healthcare professionals, regardless of their specific specialty or geographical context, must possess a strong understanding of radiation protection principles and best practices when working with ionizing radiation. The current study on the validation of the DRP‐KAPs questionnaire among Moroccan dentists aligns with this overarching perspective. Similar to the other studies, it underscores the importance of using well‐validated and reliable instruments to comprehensively evaluate radiation protection competencies among healthcare providers. These assessments can then inform the development of targeted interventions and policies to enhance radiation safety across diverse healthcare specialties. While contextual factors, such as variations in educational programs, regulations, and radiation protection practices, may exist between the different study settings, the convergence of these findings underscores the value of having validated assessment tools to promote a culture of radiation safety and optimize the use of ionizing radiation, including in the field of dental care. The robust psychometric properties demonstrated in these studies, particularly in terms of content validity and internal consistency; ensure that these instruments are comprehensive, technically accurate, and capable of generating meaningful data to guide educational interventions and policy decisions aimed at strengthening radiation safety in healthcare settings.

This study has certain limitations. The reliance on expert judgment to assess content validity introduces subjectivity and potential bias. Different experts may have different opinions about what constitutes relevant content for the DRP‐KAPs questionnaire. In addition, if the content domain is not well defined or if certain aspects are overlooked during the development phase, the questionnaire may not capture all relevant dimensions of DRP‐KAPs. This could lead to an incomplete understanding of the topic. Moreover, the limited number of experts involved in the evaluation may have impacted the CVR values, indicating the need for broader expert participation in future studies.

Although the study had a representative sample of dentists from both public and private workplaces, the samples were drawn exclusively from the Rabat‐Salé‐Kénitra region in Morocco, which limits the generalizability of the results. Data collection through self‐administered questionnaires may be susceptible to self‐reporting bias. Participants may overestimate their own KAPs regarding radiation protection, leading to inflated or inaccurate responses. This could affect the reliability and validity of the collected data.

## 5 CONCLUSION

A questionnaire was designed and developed to evaluate dentists' KAPs regarding radiation protection. This DRP‐KAPs questionnaire demonstrated adequate validity and reliability.

The development and validation of a questionnaire to assess DRP‐KAPs are crucial steps toward promoting a culture of safety and reducing radiation‐related risks in dental practice settings. By utilizing standardized questionnaires, researchers, dental professionals, and policymakers gain a comprehensive understanding of Moroccan dentists' current level of knowledge regarding radiation risks and protection, their attitudes toward radiation protection, and their actual practices in using protective measures. This holistic assessment enables stakeholders to pinpoint specific areas requiring improvement and to develop interventional programs accordingly. These programs may include continuing education to update dentists on the latest radiation safety guidelines and technologies, workshops to enhance practical skills in reducing radiation exposure during procedures, and the dissemination of best practices for radiation protection in dental practice. Future research could utilize the questionnaire to evaluate the effectiveness of educational interventions or to track changes in KAPs over time.

## AUTHOR CONTRIBUTIONS

*Conceptualization*: Naoual Elmorabit and Oum Keltoum Ennibi. *Methodology*: Naoual Elmorabit, Majdouline Obtel, Mohamed Azougagh, Asmaa Marrakchi, and Oum Keltoum Ennibi. *Validation*: Naoual Elmorabit, Mohamed Azougagh, Asmaa Marrakchi, and Oum Keltoum Ennibi. *Writing (original draft preparation; reviewing and editing)*: Naoual Elmorabit. *Verification of statistical techniques and analytical methods*: Majdouline Obtel and Naoual Elmorabit. *Manuscript drafting*: Majdouline Obtel, Mohamed Azougagh, Asmaa Marrakchi, and Oum Keltoum Ennibi. All authors have critically reviewed and approved the final draft and are responsible for the content and similarity index of the manuscript.

## CONFLICT OF INTEREST STATEMENT

The authors declare no conflicts of interest.

## ETHICAL APPROVAL

This research was ethically approved by Mohammed V University in Rabat, Faculty of Dental Medicine, the Ethics Committee for Biomedical Research (Reference number: CERB 02–22, 03/28/2022).
